# ASC Induces Apoptosis via Activation of Caspase-9 by Enhancing Gap Junction-Mediated Intercellular Communication

**DOI:** 10.1371/journal.pone.0169340

**Published:** 2017-01-05

**Authors:** Masato Kitazawa, Shigeaki Hida, Chifumi Fujii, Shun’ichiro Taniguchi, Kensuke Ito, Tomio Matsumura, Nagisa Okada, Takashi Sakaizawa, Akira Kobayashi, Michiko Takeoka, Shin-ichi Miyagawa

**Affiliations:** 1 Department of Surgery, Shinshu University School of Medicine, Matsumoto, Japan; 2 Department of Molecular Oncology, Shinshu University Graduate School of Medicine, Matsumoto, Japan; 3 Department of Molecular and Cellular Health Science, Nagoya City University Graduate School of Pharmaceutical Sciences, Mizuho-ku, Nagoya, Japan; University of South Alabama Mitchell Cancer Institute, UNITED STATES

## Abstract

ASC (apoptosis-associated speck-like protein containing a CARD) is a key adaptor molecule of inflammasomes that mediates inflammatory and apoptotic signals. Aberrant methylation-induced silencing of ASC has been observed in a variety of cancer cells, thus implicating ASC in tumor suppression, although this role remains incompletely defined especially in the context of closely neighboring cell proliferation. As ASC has been confirmed to be silenced by abnormal methylation in HT1080 fibrosarcoma cells as well, this cell line was investigated to characterize the precise role and mechanism of ASC in tumor progression. The effects of ASC were examined using *in vitro* cell cultures based on comparisons between low and high cell density conditions as well as in a xenograft murine model. ASC overexpression was established by insertion of the *ASC* gene into pcDNA3 and pMX-IRES-GFP vectors, the latter being packed into a retrovirus and subjected to reproducible competitive assays using parental cells as an internal control, for evaluation of cell viability. p21 and p53 were silenced using shRNA. Cell viability was suppressed in ASC-expressing transfectants as compared with control cells at high cell density conditions in *in vitro* culture and colony formation assays and in *in vivo* ectopic tumor formation trials. This suppression was not detected in low cell density conditions. Furthermore, remarkable progression of apoptosis was observed in ASC-introduced cells at a high cell density, but not at a low one. ASC-dependent apoptosis was mediated not by p21, p53, or caspase-1, but rather by cleavage of caspase-9 as well as by suppression of the NF-κB-related X-linked inhibitor-of-apoptosis protein. Caspase-9 cleavage was observed to be dependent on gap junction formation. The remarkable effect of ASC on the induction of apoptosis through caspase-9 and gap junctions revealed in this study may lead to promising new approaches in anticancer therapy.

## Introduction

Containing 2 death domains, caspase recruitment domains (CARD) and pyrin domains [1], the ASC protein has been shown to form aggregates in human myelocytic leukemia HL-60 cells undergoing apoptosis [2]. ASC has also been established as a key adaptor molecule of inflammasomes, activating the procaspase-1 that is necessary for processing IL-1β [3] and IL-18 [4]. Inflammasomes are critical for host defense; dysregulation of their activation contributes not only to pathogenic inflammation, but also to chronic inflammatory diseases, such as metabolic syndrome [5] and age-related disease [6]. Furthermore, inflammasome- or caspase-1-deficient mice exhibited increased tumor formation [7], and inflammasome- and IL-1β-dependent chronic inflammation contributed to the initiation and progression of cancer [8].

The *ASC* gene is known to be downregulated in human breast cancer as a result of the aberrant hyper-methylation of DNA in its promoter CpG islands [9, 10], which has since been documented in various cancers. In our previous study, silenced *ASC* was re-expressed by treatment with the DNA methyltransferase inhibitor 5'-aza-2'-deoxycytidine (5'-aza-dC) in methylation-positive human melanoma [11] and colorectal cancer [12] cell lines. This epigenetic inhibition of *ASC* in cancer cells implied a possible role as a tumor suppressor gene [13].

Thereafter, numerous studies have demonstrated an inhibitory effect of ASC on tumorigenesis. Colorectal cancer was enhanced upon genetic deletion of caspase-1 or ASC [14], while ASC-overexpressing lymphoma cells showed reduced metastasis [15]. The understanding of the mechanisms of ASC has progressed as well, with reports of tumorigenesis inhibition in primary melanoma via ASC expression by restricting NF-κB activity [16] and decreased P53- and p21-related cell apoptosis by knockdown of ASC [17].

Intercellular communication halts normal cell proliferation by cell cycle arrest when cells reach a high density in culture conditions. However, this cell contact inhibition is frequently impaired in tumor cells, resulting in abnormal proliferation [18]. Several signaling pathways, including those of p53 [19], p21 [20], cadherin [21], and mTOR and p27 [22], have been studied to address this phenomenon. The present study turned to the role of ASC in this aberrant viability at a high cell density with a focus on apoptosis and gap junctions, i.e., intercellular communication-dependent programmed cell death, in the HT1080 malignant phenotype human fibrosarcoma cell line. Gap junctions provide a direct route for metabolites and signaling molecules to pass from cell to cell. As decreased expression of gap junction-related molecules inhibited intercellular communication in many cancer cell lines [23, 24], dysregulation of junctional communication might play a critical role of cancer development.

The ASC-dependent apoptosis was elicited by the activation of caspase-9 and suppression of NF-κB-related X-linked inhibitor-of-apoptosis protein (XIAP) in a gap junction-mediated fashion. Moreover, reproducible competitive assays using FACS analysis based on internal controls were established for the precise evaluation of cell viability.

## Materials and Methods

### Cell culture

Cells from the HT1080 Human fibrosarcoma cell line, HT1080, was obtained from the IFO Animal Cell Bank (Osaka, Japan) and cultured in Dulbecco’s modified Eagle’s medium supplemented with 10% fetal bovine serum at 37°C in 5% CO_2_.

### Quantitative reverse-transcription polymerase chain reaction (RT-PCR)

Total RNA was extracted with NucleoSpin RNAII (Takara Bio, Otsu, Japan), and RT was performed with PrimeScript^®^ RT Master Mix (Takara Bio). The PCR was set up with SYBER Premix Ex Taq^™^ II (Takara Bio) and carried out on a Thermal Cycler Dice Real Time System II device (Takara Bio). The sequences of the specifically designed primers are listed in Table 1.

**Table 1:** Primer sequences used for quantitative RT-PCR.

Gene name	Primer sequence (5'-3')
β-actin	F: GGACTTCGAGCAAGAGATGG
	R: GTGGATGCCACAGGACTCCAT
ASC	F: CTCCTCAGTCGGCAGCCAAG
	R: ACAGAGCATCCAGCAGCCAC
p53	F: TTCGACATAGTGTGGTGGTGC
	R: GCCCATGCAGGAACTGTTACAC
p21	F: AGGTGGACCTGGAGACTCTCAG
	R: GCTTCCTCTTGGAGAAGATCAGC
IL-1β	F: ACAGATGAAGTGCTCCTTCCA
	R: GTCGGAGATTCGTAGCTGGAT
MMP-9	F: TGACGAGTTGTGGTCCCTGG
	R: AGGAGCGGCCCTCGAAGATGAAG
VEGF	F: CGAAACCATGAACTTTCTGC
	R: CCTCAGTGGGCACACACTCC
XIAP	F: TGGTTGCAGATCTAGTGAATGCTC
	R: CGCCTTAGCTGCTCTTCAGTAC
Connexin 43	F: TTCATGCTGGTGGTGTCCTTG
	R: GCTCTTTCCCTTAACCCGATCC

### Western blotting analysis

Cells were lysed in lysis buffer containing 20 mM Tris-HCl pH 7.5, 150 mM NaCl, 0.5% deoxycholic acid, 1% NP-40, 2 mM EDTA, 1% SDS, 1 mM phenylmethyl sulfonylfluoride, 1 mM sodium orthovanadate, 25 mM NaF, and 1x complete protease inhibitor cocktail (Roche, Mannheim, Germany) for 30 minutes at 4°C. The lysates were separated by 15% SDS-PAGE. The anti-human ASC mAb described previously ([2]; MBL, Nagoya, Japan) and anti-human caspase-9 mAb (Cell Signaling Technology, Beverly, MA) were adopted in Western blotting assays.

### Transfection of plasmid vectors and establishment of stable clones

Human full-length *ASC* genes were inserted into pcDNA3 vectors (Invitrogen, Carlsbad, CA). Empty pcDNA3 vectors (control) and *ASC*/pcDNA3 vectors (ASC) were transduced into HT1080 cells with GeneJuice (Novogen, San Diego, CA). G418-resistant clones were selected at 14 days of incubation.

### Cell viability and colony formation assays

For *in vitro* viability assays, 3x10^4^ of control or ASC-expressing cells were seeded onto 60 mm plates for low cell density culture conditions or onto 12-well plates for high cell density culture conditions. In colony formation assays, approximately 500 cells were seeded onto 100 mm plates and incubated for 11 days. Colonies larger than 0.25 mm^2^ were counted using NIH Image J software.

### Tumorigenesis *in vivo*

To examine tumorigenesis *in vivo*, 1x10^6^ of control or ASC-expressing cells were subcutaneously injected into the flanks of 7-week-old male BALB/c nude mice (CLEA Japan, Tokyo, Japan). Tumor volume was evaluated using the formula of: volume (mm^3^) = 0.5 x width^2^ (mm) x length (mm). All mice were sacrificed by means of sodium pentobarbital anesthesia on day 12. Experiments were carried out in accordance with the Guidelines for Animal Care and Experimentation of Shinshu University School of Medicine.

### TUNEL and immunohistochemistry

*In situ* detection of DNA fragmentation in tumor tissues was performed using TUNEL staining with *in situ* apoptosis detection kits (Takara Bio). For the staining of Ki-67 (mAb, DAKO, Kyoto, Japan), antigen retrieval was done by boiling sections in a microwave oven in 0.01 M citric acid, pH 6.0. The numbers of TUNEL-and Ki-67-positive cells were counted in random high-power fields.

### Retroviral transduction

For retroviral transduction, the *ASC* gene was inserted into pMX-IRES-GFP vectors (Cell Biolab, Inc., San Diego, CA), which were then transfected into amphotropic packaging cells using GeneJuice (Novogen). The virus-containing supernatants were harvested at 24 h and 48 h. Retrovirus infection was performed on RetroNectin (Takara Bio)-coated plates. Transduction efficiency was confirmed in terms of GFP-positive ratio by flow cytometry (FACS, BD FACS Canto II, Becton Dickinson, Franklin Lakes, NJ), and the early-passaged cells were used in subsequent experiments.

### Competitive assays

Competitive assays were conducted using parental cells as an internal control for enhanced objectivity and reproducibility. After retroviral transduction with pMX-IRES-GFP vectors, vector-transduced HT1080 cells (GFP-positive) and non-treated parental cells (GFP-negative) were mixed at approximately equal amounts. For low cell density assays, 3x10^4^ of mixed cells were seeded onto 12-well plates on day 0, i.e., 1.5x10^4^ of parental cells and 1.5x10^4^ of pMX-IRES-GFP vector-transfected cells (control) or 1.5x10^4^ of pMX-IRES-GFP/*ASC* vector-transfected cells (ASC-expressing). For high cell density assays, 3x10^5^ of mixed cells were seeded onto 12-well plates in an identical fashion. The ratio of GFP-positive cells to parental cells was determined by FACS and defined as “GFP-positive cell ratio”. Then, “relative GFP-positive ratio (%)” was calculated as: 100 x (GFP-positive cell ratio on target day / GFP-positive cell ratio on day 0).

### Annexin V and 7-AAD staining

For evaluation of cell death, cells were harvested and resuspended in Annexin V Binding Buffer (BioLegend, San Diego, CA) followed by Alexa Fluor 647-conjugated annexin V (BioLegend) for 15 min at room temperature. Cells were further resupended in Annexin V Binding Buffer dissolved with 7-AAD (BioLegend). The double-positive percentage of annexin V and 7-AAD was analyzed by FACS.

### Knockdown of p53, p21 and connexin 43

For knockdown of the retroviral-mediated target genes p53, p21, and connexin 43, shRNA sequences were ligated into pSINsi-hU6 or pSINsi-DK I vectors (Takara Bio) as listed in Table 2. G418-resistant cells were selected at 14 days of incubation. Knockdown efficiencies were confirmed by quantitative RT- PCR analysis.

**Table 2:** Target sequences for shRNA.

Target gene	Vector	Promoter	Targeted sequence (5'-3')
Scramble	pSINsi-hU6	U6	TCTTAATCGCGTATAAGGC
p53	pSINsi-hU6	U6	GACTCCAGTGGTAATCTAC
p21	pSINsi-hU6	U6	TCACTGTCTTGTACCCTTGT
Connexin 43	pSINsi-DK I	U6	Cx43DNA1: TGAGTACCACCTCCACCGG
			Cx43DNA2: TAAATACCAACATGCACCT

### Statistical analysis

Statistical significance was evaluated using the Student’s *t*-test on the data of 3–8 experiments for each assay. A p value of <0.05 was accepted as statistically significant. All values were expressed as the mean ± standard error of the mean.

## Results

### ASC suppressed cell viability in high, but not low, cell density conditions

**Fig 1:**
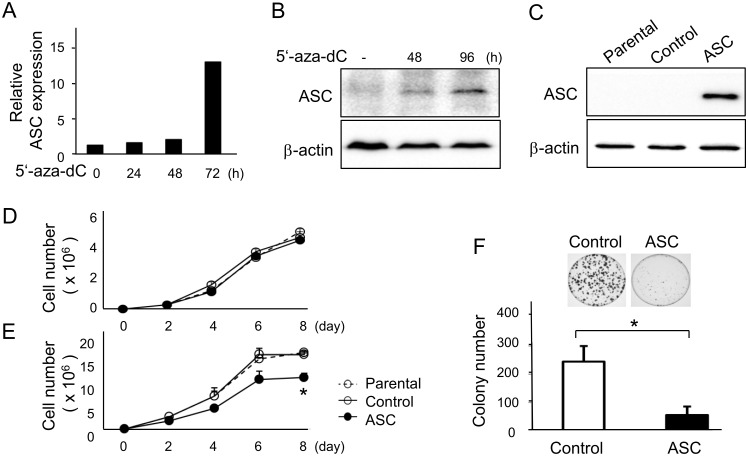
5'-aza-dC treatment restored ASC expression and overexpression of ASC suppressed cell viability in high cell density cultures. HT 1080 cells were treated with 8μM 5'-aza-dC, and expression of ASC was detected by (A) quantitative RT-PCR (n = 2) and (B) Western blotting. (C) Expression of ASC inserted into pcDNA3 vectors and stably transfected into cells was confirmed by Western blotting. Viability of ASC-expressing and control cells at (D) a low cell density (n = 8), (E) a high cell density (n = 8), and (F) in colony formation assays (n = 3). *p<0.05.

As the expression of ASC was found to be decreased in several progressive cancer cell lines by aberrant methylation, we treated HT1080 cells with the demethylating reagent 8μM 5**'**-aza-dC (Sigma-Aldrich, St. Louis, MO) and observed that demethylation increased the expression of *ASC* mRNA by 12.8-fold (Fig 1A) in addition to ASC protein level (Fig 1B) in repeated experiments, indicating that the *ASC* gene was indeed silenced by methylation in HT1080 cells. Thereafter, we established stably transfected HT1080 clones expressing ASC for investigation of the effects of ASC on cell viability and colony-forming activity *in vitro*. The expression of transfected ASC introduced into pcDNA3 vectors was confirmed by Western blotting (Fig 1C).

When cells were plated at a low cell density, the viability rate was similar between ASC-expressing and control vector-transfected cells (Fig 1D). In contrast, the viability of ASC-expressing cells was significantly suppressed on day 8 as compared with control cells in a high density culture (Fig 1E). Furthermore, the number of colonies, which mimicked the *in vivo* environment, was significantly smaller for ASC-expressing cells (Fig 1F). These results indicated that ASC suppressed cell viability at a high cell density, presumably through close interactions with neighboring cells, but not at a low density.

### ASC negatively regulated tumorigenesis *in vivo*

**Fig 2:**
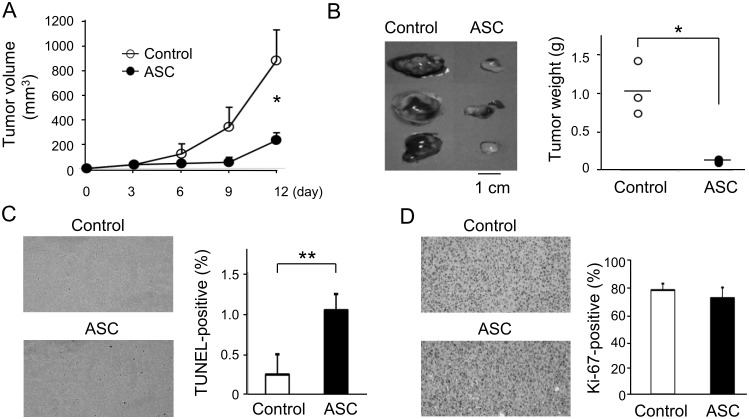
ASC negatively regulated tumorigenesis *in vivo*. (A) Tumor volumes of ASC-expressing and control cells subcutaneously inoculated into the flanks of immunodeficient mice (n = 3). (B) Tumor weights of mice sacrificed on day 12. Staining and number of (C) TUNEL-positive apoptotic cells (n = 6) and (D) Ki-67-positive cells (n = 6). *p<0.05, **p<0.01.

We next examined the effects of ASC on tumorigenicity *in vivo*. As shown in Fig 2A, the tumor volume of ASC-expressing cells was significantly smaller than that of control cells on day 12. Tumor weight was also significantly lower in ASC-expressing cells (Fig 2B). The number of TUNEL-positive apoptotic cells in ASC-transfected tumors was increased by 4-fold as compared with that of control tumors (Fig 2C), while no significant differences were observed for Ki-67 expression (Fig 2D). These results suggested that ASC regulated tumor formation by the induction of apoptosis rather than by cell cycle retardation.

### Confirmation of the suppressive effect of ASC on cell viability by competitive assays

**Fig 3:**
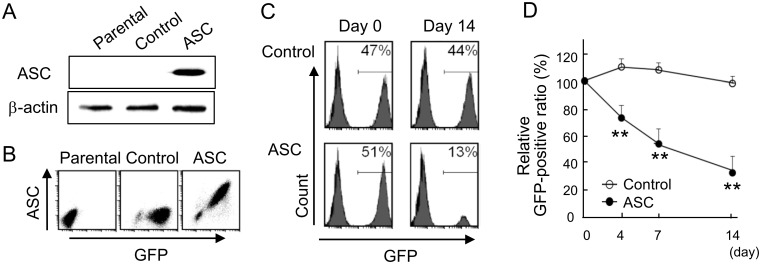
Competitive assays using retroviruses containing vectors bicistronicly encoded with GFP and ASC. (A) Expression of ASC inserted into pMX-IRES-GFP vectors was confirmed by Western blotting. (B) A correlation of ASC with GFP was disclosed by FACS. (C) Population of GFP-positive cells simultaneously expressing ASC on day 14 as detected by FACS. (D) Time course of relative GFP-positive ratio in a high cell density culture (n = 4). **p<0.01.

Retrovirus-expressing bicistronic message-encoding ASC and GFP were prepared for competitive assays. ASC transduction was detected by Western blot analysis (Fig 3A), and a positive correlation ASC with GFP was confirmed by FACS analysis (Fig 3B). The cells were transfected with greater than 90% efficiency by the retroviral vector. GFP-transduced cells did not exhibit markedly altered growth kinetics or GFP-positive cell ratio.

The GFP-positive cell ratio was expected to decrease in ASC-expressing cells via an inhibitory effect on viability. This was evident by FACS on day 14 in high cell density cultures, indicating that ASC suppressed cell viability (Fig 3C). The relative GFP-positive ratio was significantly decreased in ASC-expressing cells as compared with control cells at a high cell density as well (Fig 3D). Such findings confirmed that the competitive mix culture was a reproducible and reliable method for the evaluation of cell viability, which was therefore employed for subsequent experiments. Here, GFP-positive cell ratio was the ratio of GFP-positive cells to parental cells, after which relative GFP-positive ratio (%) was calculated as: 100 x (GFP-positive cell ratio on target day / GFP-positive cell ratio on day 0), as described in the Materials and Methods.

### ASC-mediated apoptosis, but not necrosis, was induced in high cell density conditions

**Fig 4:**
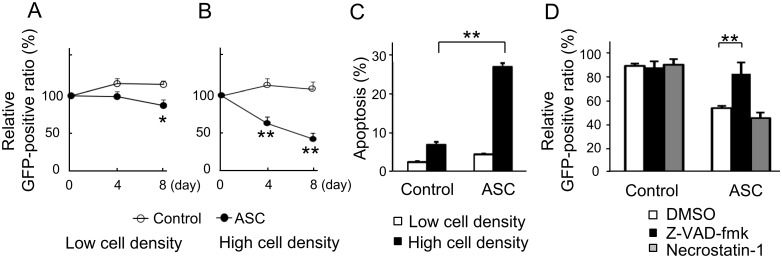
ASC-mediated apoptosis, but not necrosis, was induced in high cell density conditions. Comparison of relative GFP-positive ratio between control and ASC-expressing cells at (A) a low cell density and (B) a high cell density (n = 6 for each). (C) Percentage of annexin V/7-AAD double-positive cells as detected by FACS. (D) Treatment of cells with a pan-apoptosis inhibitor (Z-VAD-fmk) or inhibitor of necrosis (Necrostatin-1) at a high cell density (n = 4). *p<0.05, **p<0.01.

ASC suppressed relative GFP-positive ratio more readily at a high cell density as compared with at a low cell density (Fig 4A and 4B). The status of apoptosis was examined with annexin V/7-AAD using FACS at 4 days after inoculation. At a low cell density, the cell populations undergoing apoptosis were minimal, and there was no significant difference between ASC-expressing and control cells (Fig 4C). On the other hand, at a high cell density, ASC-expressing cells had significant, 3-fold increased percentages of annexin V/7-AAD double-positive cells, i.e., late apoptosis or necrotic cells, as compared with control cells (Fig 4C).

We next sought to distinguish the precise factor causing cell death as either apoptosis or necrosis. An inhibitor of RIP-1-dependent necrosis (Necrostatin-1, BioMol, Plymouth Meeting, PA) failed to abrogate ASC-dependent cell death, whereas a pan-caspase inhibitor (Z-VAD-fmk, MBL) almost completely blocked ASC-dependent cell death (Fig 4D). These results indicated that the reduced number of ASC-expressing cells was associated with an increase in apoptosis, and not necrosis, which was consistent with our *in vivo* experiments.

### ASC-mediated apoptosis induced at a high cell density was independent of p53 and p21 function

**Fig 5:**
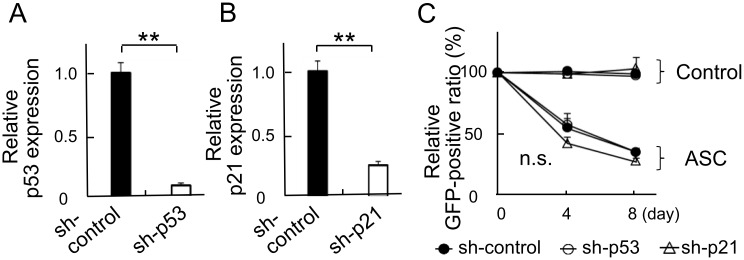
Knockdown of p53 or p21 with shRNA did not affect ASC-dependent apoptosis at a high cell density. Expression of (A) p53 and (B) p21 was confirmed by quantitative RT-PCR (n = 6 for each). (C) Effect of p53 or p21 knockdown on control and ASC-expressing cell relative GFP-positive ratio (n = 6 for each). **p<0.01, n.s.: not significant.

To determine if p53 or p21 function was required for ASC-induced apoptosis at a high cell density, we generated p53- or p21-silenced HT1080 cells by retroviral shRNA-mediated knockdown that was confirmed by quantitative RT-PCR (Fig 5A and 5B). Unexpectedly, knockdown of neither p53 nor p21 attenuated ASC-dependent apoptosis in viability assays (Fig 5C), indicating that the apoptosis induced by ASC at a high cell density occurred in a p53- and p21-independent manner.

### Gap junction-mediated activation of caspase-9 promoted ASC-dependent apoptosis at a high cell density

**Fig 6:**
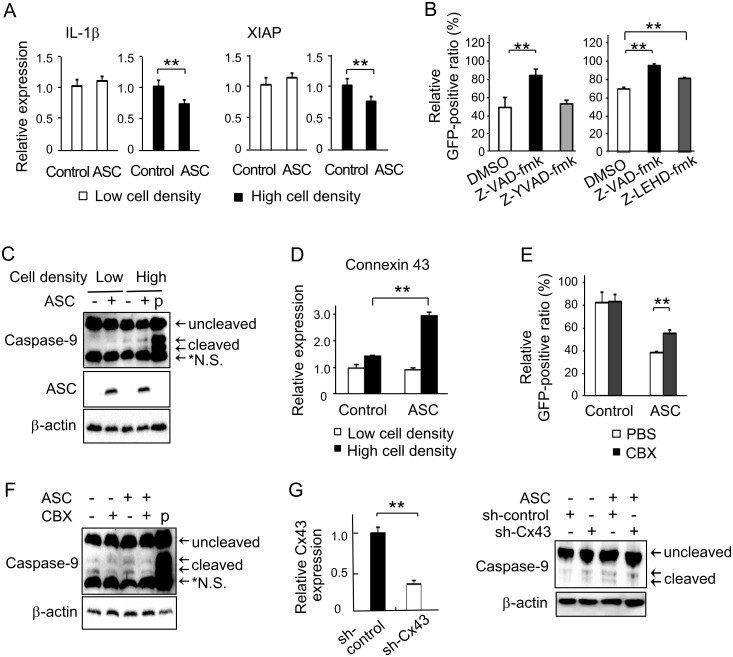
Involvement of caspase-9 and gap junctions on ASC-dependent apoptosis at a high cell density. (A) Relative mRNA expressions of the NF-κB-regulated genes IL-1β and XIAP were evaluated by quantitative RT-PCR at both low and high cell densities (n = 4 for each). (B) Effects of caspase inhibitors on ASC-dependent apoptosis as evaluated by relative GFP-positive ratio at a high cell density. Z-VAD-fmk; pan-caspase inhibitor, z-YVAD-fmk; caspase-1 inhibitor, Z-LEHD-fmk; caspase-9 inhibitor (n = 4 for each). (C) Activation of caspase-9 induced by ASC introduction as detected by Western blotting at low and high cell densities. (D) Relative mRNA expression of connexin 43 evaluated by quantitative RT-PCR at both low and high cell densities (n = 3 for each). (E) Effect of the gap junction inhibitor CBX on relative GFP-positive ratio at a high cell density (n = 4). (F) Effects of CBX on the activation of caspase-9 at a high cell density by Western blotting. (G) Confirmation of connexin 43 (Cx 43) silencing performed by quantitative RT-PCR (n = 3). And effects of connexin 43 silencing on the activation of caspase-9 at high cell density by Western blotting. Representative results of triplicate experiments. **p<0.01.

Since NF-κB has been shown to correlate with ASC and apoptosis, the involvement of NF-κB-related molecules was investigated to elucidate the pathway of the ASC-dependent apoptosis caused in high cell density conditions. Quantitative RT-PCR analysis demonstrated that the expressions of the NF-κB-related genes *IL-β*and *XIAP* were significantly suppressed in ASC-expressing cells as compared with controls at a high cell density (Fig 6A). No such difference was observed at a low cell density. The induction of ASC has been documented to activate caspase-1 and process pro IL-1β [25], while XIAP was reported to inhibit the activation of caspase-9 [26]. Therefore, caspase-1 and caspase-9 were judged to be candidates involved in the ASC-dependent apoptosis in high cell density conditions.

A pan-caspase inhibitor (Z-VAD-fmk, MBL) blocked ASC-dependent cell death. An inhibitor of caspase-9 (Z-LEHD-fmk, MBL) significantly abrogated ASC-dependent cell death as well, but a caspase-1 inhibitor (Z-YVAD-fmk, MBL) did not produce any marked alterations (Fig 6B). These results indicated that the ASC-facilitated apoptosis was at least partially mediated by caspase-9. We subsequently analyzed the potential activation of endogenous caspase-9 in response to ASC induction. The protein level of cleaved caspase-9 in ASC-expressing cells was remarkably higher than that in controls at a high cell density, but not at a low one; caspase-9 cleavage was hardly detectable in low cell density conditions (Fig 6C).

Lastly, since the loss of gap junction-mediated intercellular communication has been shown to facilitate tumorigenesis and tumor cell survival, we investigated the expression of connexin involved in gap junction assembly. The mRNA expression of connexin 43 was slightly increased at a high density in control cells, while significantly increased connexin 43 mRNA expression was observed in ASC-expressing cells at a high density (Fig 6D). We further analyzed the role of gap junctions in ASC-dependent apoptosis via the evaluation of caspase-9 cleavage. The gap junction inhibitor carbenoxolone (CBX; Sigma-Aldrich) significantly attenuated the growth inhibitory effects of ASC (Fig 6E) and the cleavage of caspase-9 in ASC-expressing cells (Fig 6F). As CBX was not specific for gap junctions, we also performed knockdown of connexin 43 to disassemble gap junctions. Connexin 43 was confirmed to be effectively silenced (Fig 6G). Cleaved caspase-9 was observed in ASC-expressing cells at a high cell density, which was prevented in ASC-expressing, connexin 43-silenced cells. These results inferred that suppression of cell growth by ASC at a high density at least partially depended on gap junctional communication.

## Discussion

After observing that the viability of HT1080 fibrosarcoma cells was suppressed by ASC when in close proximity to neighboring cells, we examined several signaling pathways to reveal that ASC suppressed XIAP expression, enhanced the involvement of gap junctions, and consequently activated caspase-9, which resulted in the induction of apoptosis, and not necrosis, in HT1080 fibrosarcoma cells via close interactions with adjacent cells in high density conditions.

In the murine *in vivo* experiment, the suppression of tumorigenesis by ASC induction was suggested to occur by apoptosis rather than cell cycle arrest. Therefore, we focused on programmed cell death and investigated its characteristics in *in vitro* experiments based on comparisons between low and high cell density conditions. ASC-expressing cells exhibited increased apoptosis at a high cell density. ASC has been reported to play an essential role in the intrinsic mitochondrial pathway of apoptosis through a p53-Bax network [27], with the anti-tumorigenic function of ASC being partially regulated by the activation of p53 and p21 signaling [17]. However, experiments with shRNA revealed that the ASC-dependent apoptosis induced at a high cell density in this study was independent of p53 and p21.

Erl et al. described that NF-κB inhibition increased cell death at a low cell density, while cells in high density cultures exhibited high NF-κB activity and were insensitive to the induction of apoptosis [28]. Forced expression of ASC was seen to enhance NF-κB activity in metastatic melanoma but inhibit NF-κB in primary melanoma [16], indicating that the effects of ASC on NF-κB depended on the degree of malignancy and cellular context. Based on these studies, we investigated the effects of ASC on several NF-κB-related genes and witnessed that ASC inhibited the induction of the NF-κB-dependent genes IL-1β and XIAP at a high cell density. IL-1β is a major target of caspase-1 and XIAP has been reported to regulate the activity of caspase-9 and inhibit apoptosis [29]. Here, trials with z-YVAD-fmk, an inhibitor of caspase-1, and z-LEHD-fmk, an inhibitor of caspase-9, revealed that ASC-dependent apoptosis in high cell density conditions was likely mediated by the cleavage of caspase-9 only.

ASC has been reported to trigger Fas ligand-induced caspase-8-dependent apoptosis [30, 31], but reports on ASC and caspase-9 are few. McConnell et al. [32] demonstrated that ASC (TMS1)-induced apoptosis proceeded through a CARD-dependent aggregation step followed by the activation of a caspase-9-mediated pathway. In general, death-inducing signaling complexes have been identified as activators of caspase-8 [33, 34], while apoptosomes provide the activation platform for initiation of caspase-9 [29, 35]. A recent study [36] revealed that caspase-9 was activated allosterically by binding to apoptosomes, in which a platform was assembled in response to mitochondria-dependent apoptosis under the influence of XIAP. In the inflammatory response, spinal cord injury activates inflammasomes containing ASC and leads to the cleavage of XIAP [37]. However, reports on this phenomenon are scarce, with even fewer related to tumors. It was intriguing that ASC activated caspase-9 with suppression of XIAP and resulted in apoptosis in a high cell density culture only.

We ensuingly addressed how increased cell density could possibly trigger caspase-9-activated apoptosis. Apoptosis was found to be induced at a high cell density in relation to TGF-β and Bcl-2 in HL-60 cells [37]. Cell-cell contact promoted the nuclear translocation of p21/Waf-1-induced cellular growth arrest and increased apoptosis [38]. Elsewhere, p53 activation was suppressed in density-plated cells [19]. An involvement of p21 or p53 in ASC-dependent apoptosis at a high cell density was excluded earlier in our experiment.

Cells constitutively interact with each other, and cell-cell communication through gap junction formation plays an imperative role in tumor cell survival and death. The loss of gap junctions assembled by connexins was reported to facilitate tumorigenesis in prostate [23] and liver [24] cancers. Therefore, we inhibited gap junction establishment by CBX or connexin 43 knockdown to elucidate its functions in apoptosis. They reduced the cleavage of caspase-9 and growth suppression by ASC induction, indicating that gap junctions were possible mediators of ASC-dependent apoptosis in HT1080 tumor cells in high cell density conditions. However, findings in a single cell line may not always be extrapolated to other cell types. According to Larson et al. [39], connexin 43 mRNA levels were high in subconfluent conditions but decreased at confluency in normal endothelial cells. Slightly increased mRNA amounts were, however, observed in our control cells, the level of which became significantly increased in ASC-expressing cells at a high density. Gap junction assembly by connexin 43 was documented to propagate cell-killing signals initially generated by a single cell spontaneously initiating apoptosis to surrounding cells in a bladder carcinoma cell line [40]. Since the expression of connexin 43 is regulated by the PI3K/AKT/mTOR and Mnk1/2 pathways [41], the role of ASC in gap junction signaling that occurs at a high density needs to be clarified.

It is noteworthy that the present study employed competitive assays in addition to stable clone assays. Since parental cells were mixed as an internal control and the ratio of transfectants to parental cells was evaluated by FACS, the competitive assays were believed to be relatively more objective and reproducible than conventional set-ups. Competitive assays also avoid the risk of selecting champion clones that limits the reliability of stable clone assays.

In conclusion, the remarkable effects of ASC on the induction of apoptosis through caspase-9 activation and gap junctions may have important bearings in tumor suppression and represent a promising new target in anticancer therapy.
